# Hospital in Motion, a Multidimensional Implementation Project to Improve Patients’ Physical Behavior During Hospitalization: Protocol for a Mixed-Methods Study

**DOI:** 10.2196/11341

**Published:** 2019-04-09

**Authors:** Lotte Martine Maria van Delft, Petra Bor, Karin Valkenet, Cindy Veenhof

**Affiliations:** 1 Department of Rehabilitation, Physiotherapy Science and Sport Brain Center Rudolf Magnus University Medical Center Utrecht, Utrecht University Utrecht Netherlands

**Keywords:** hospitalization, implementation science, activities of daily living, interdisciplinary care, mobility, physical activity

## Abstract

**Background:**

Despite the evidence of the adverse consequences of immobility during hospitalization, patients spend most of the time in bed. Although physical activity is a modifiable factor that can prevent in-hospital functional decline, bed rest is deeply rooted in the hospital culture. To attack this, a multidimensional approach is needed. Therefore, Hospital in Motion, a multidimensional implementation project, was designed to improve physical behavior during hospitalization.

**Objective:**

The primary objective of this study is to investigate the effectiveness of Hospital in Motion on inpatient physical behavior. Secondary objectives are to investigate the effectiveness on length of hospital stay and immobility-related complications of patients during hospitalization and to monitor the implementation process.

**Methods:**

For this study, Hospital in Motion will be implemented within 4 wards (cardiology, cardiothoracic surgery, medical oncology, and hematology) in a Dutch University Medical Center. Per ward, multidisciplinary teams will be composed who follow a step-by-step multidimensional implementation approach including the development and implementation of tailored action plans with multiple interventions to stimulate physical activity in daily care. A prepost observational study design will be used to evaluate the difference in physical behavior before and 1 year after the start of the project, including 40 patients per time point per ward (160 patients in total). The primary outcome measure is the percentage of time spent lying, measured with the behavioral mapping method. In addition, a process evaluation will be performed per ward using caregivers’ and patient surveys and semistructured interviews with patients and caregivers.

**Results:**

This study is ongoing. The first participant was enrolled in October 2017 for the premeasurement. The postmeasurements are planned for the end of 2018. The first results are expected to be submitted for publication in autumn 2019.

**Conclusions:**

This study will provide information about the effectiveness of the Hospital in Motion project on physical behavior and about the procedures of the followed implementation process aimed to incorporate physical activity in usual care. These insights will be useful for others interested in changing physical behavior during hospitalization.

**Trial Registration:**

Netherlands Trial Register NTR7109; https://www.trialregister.nl/trial/6914 (Archived by WebCite at http://www.webcitation.org/76dyhdjdd)

**International Registered Report Identifier (IRRID):**

DERR1-10.2196/11341

## Introduction

### Background

More than 2 million patients are admitted to Dutch hospitals yearly, with a mean admission time of 7 days [1]. Although hospital admissions are necessary to diagnose or treat patients for health issues, hospital admissions also have downsides. Diverse studies show that hospitalized patients spend most of the time lying in bed, whereas in the last 20 years, a growing body of evidence is established showing the adverse consequences of bed rest [2,3]. Restricted physical activity and immobilization can increase hospital-related complications [3,4], and many studies have proven that inactivity is associated with reduced muscle mass and strength [5]. In addition, bed rest results in an increased risk of diverse medical complications [6-8]. Moreover, lower levels of physical activity are associated with a functional decline and new disability in activities of daily living (ADL) after discharge [3,4,9-12]. This functional decline is labeled as a hospitalization-associated disability (HAD), and HADs have profound implications for patients as it leads to long-term care in nursing homes, readmissions, and even death [11]. In research reports, HADs are described as both preventable and iatrogenic and as a direct result from the actions of a health care provider or institution. HADs can, therefore, be considered as collateral damage of the treatment in a hospital in which health care professionals and policy makers have a responsibility in resolving this problem [13]; especially, as early mobilization and higher levels of physical activity during hospitalization have proven to decrease the risk of complications and length of stay (LOS) [14].

Nevertheless, patients are reflexively put into pajamas, transferred into bed [15], and spend less than 6% of the day being active [2-4,9]. Lack of knowledge and time is often mentioned by caregivers as a barrier to promote physical activity [16,17]. This lack of time results in nurses prioritizing their medical tasks above assisting with patient mobilization and stimulating physical activity in patients with the ability to perform their own ADL tasks [16,17]. Studies targeting sedentary behavior during hospitalization have shown that physical activity is a modifiable factor that can prevent in-hospital functional decline [14,18-20]. These studies mostly focused on single interventions, whereas sedentary behavior is deeply rooted in the hospital culture. A multidimensional project focusing on environment, caregivers, and patients using multiple interventions may possibly be even more effective [21]. Even so, literature suggests that a comprehensive and flexible framework may help create sustainable interventions, leading to significant changes in clinical practice [22]. However, projects or studies to improve physical behavior focusing on the whole system, integrating physical activity in all levels of daily hospital care, are not common. Moreover, these studies focused mainly on elderly, whereas low mobility is of all ages [19,22]. Therefore, Hospital in Motion, a multidimensional project to improve patients’ physical behavior during hospitalization, has been developed.

### Objectives

The primary objective of this study is to investigate the effectiveness of Hospital in Motion on physical behavior within 4 wards (cardiology, cardiothoracic surgery, medical oncology, and hematology).

Secondary objectives are to investigate the effectiveness on length of hospital stay and immobility-related complications of patients during hospitalization and to monitor the implementation process.

## Methods

### Context

In November 2015, the project Hospital in Motion was started at the University Medical Center Utrecht (UMC Utrecht). Hospital in Motion is a complex multidimensional project primarily designed to improve physical behavior during hospital stay, defined as a decrease in patients’ sedentary behavior (lying) and increase in physical activity (ie, standing, walking, and exercising). This project follows 2 approaches. The first approach focusses on creating a hospital-wide awareness of the high amount of sedentary behavior during the hospital stay and the known associated adverse effects, and the necessity to incorporate physical activity in usual care. The second approach includes the development and implementation of tailored action plans for each clinical ward. In 2016 and 2017, a pilot study was performed on the geriatric department. Preliminary results and gained experiences during this pilot form the basis of this study protocol.

### Setting

This study will be conducted within 4 wards (cardiology, cardiothoracic surgery, medical oncology, and hematology) of the UMC Utrecht, the Netherlands. Per ward, a tailored action plan will be implemented. The study protocol was assessed and approved by the medical ethics committee of the UMC Utrecht (study protocol number 16-250). Verbally informed consent was obtained from all patients.

### Study Design

**Figure 1:**
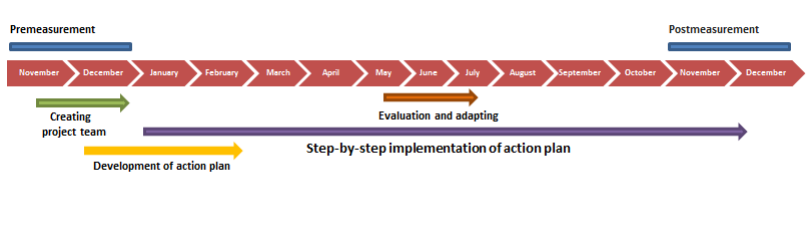
Timeline of the implementation project Hospital in Motion.

An observational study with a prepost design will be used to evaluate the effectiveness of Hospital in Motion on physical behavior. In addition, the implementation process will be evaluated by using a qualitative approach. Data will be collected before and after implementation. The duration of the implementation project is planned for 10 months, starting in January 2018 (Figure 1).

### Implementation Approach and Interventions

**Figure 2:**
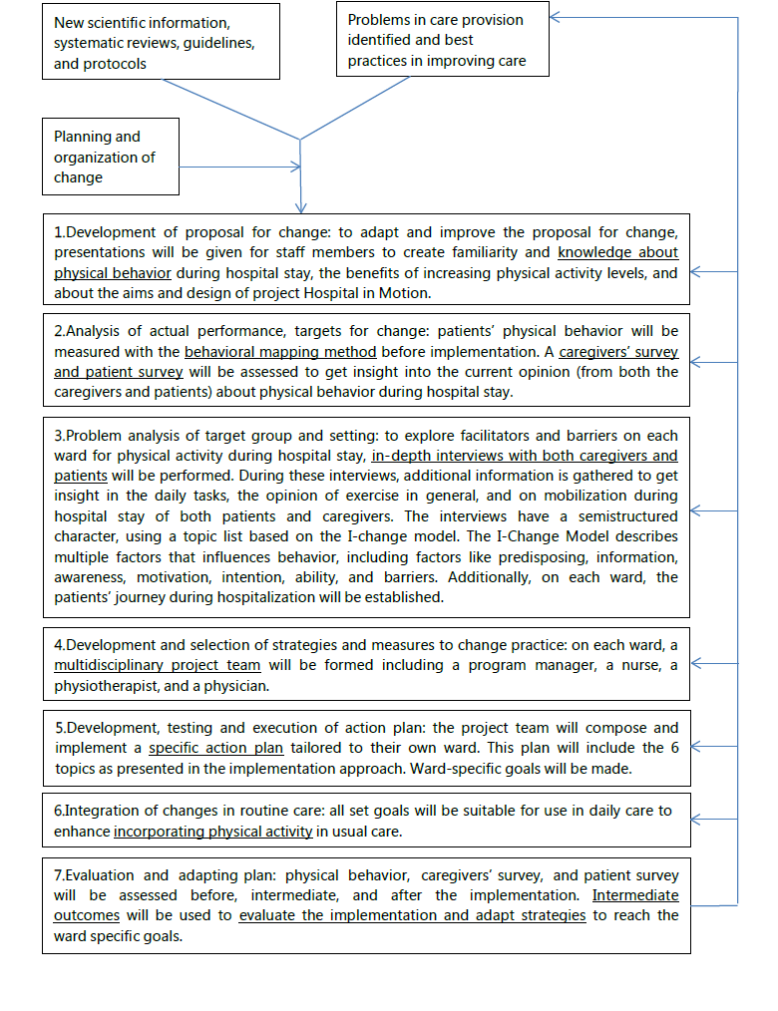
Implementation model based on the study by Grol and Wensing.

Hospital in Motion will be implemented following the step-by-step model of Grol and Wensing (Figure 2) [23]. Steps 1 to 3 include the development of proposal for change, analysis of actual performance, and problem analysis. Step 4 includes the selection of strategies and measures to change practice, which will be identified by a multidisciplinary project team per ward. During step 5, an action plan consisting of multiple interventions will be developed, tested, and executed at each ward. This plan will consist of 6 general topics:

1. Education: Education is an important cornerstone for increasing awareness on the importance of physical activity [17,24], for example, education for the staff members about the dangers of bed rest and posters and leaflets for patients about the importance of staying active during hospital stay.
2. Physical activity as part of usual care: For successful implementation, physical activity needs to be incorporated in usual care and all caregivers with direct patient contact need to be involved [17,25], for example, integrating questions on the physical activity level in the anamneses of nurses and physicians, standardized reporting of daily mobility levels in the patient records, and discussing the patients mobility during multidisciplinary meetings.
3. Involving third parties: Involving the social environment (ie, family, friends, or volunteers) to improve inpatient physical behavior, for example, family and visit leaflets with information about the importance of physical activity during hospitalization and tips to improve patients’ physical activity [26,27].
4. Stimulating environment: Currently, hospital wards are not stimulating environments for performing physical activity [28]. Changes in the environment are conditional for stimulating physical activity, for example, by adjustments of the accommodation inpatient areas, introducing shared lunching, and visualizing walking routes.
5. Mobilization milestones: Daily mobilization goals are successful in increasing walking distance, ADL activities, and number of mobilization moments out of bed [14]. The use of a mobility scale or activity trackers are examples of interventions, which could be used to set personal mobility goals.
6. Technology support: Implementing technological applications such as cycle ergometers with interactive screens, activity trackers, or mobile apps to support, stimulate, and measure physical activity [29].

### Outcome Evaluation

In total, 160 patients will be included during a period of 2 months (40 patients per ward). Each patient admitted in the specific ward is eligible to participate in this study. Exclusion criteria for participating in this study were delirium and other cognitive impairments, whereby patients who were not able to provide informed consent were excluded. Patients receiving terminal care were also excluded.

#### Primary Outcome

Physical behavior will be measured with the behavioral mapping method [30] and will be assessed before and after the implementation period (Figure 1). Patients will be observed on a random weekday of their stay in a fixed order every 10 min for 1 min. During this minute, the patients’ location, body position, daily activity, and direct contact will be registered [30]. A maximum of 8 patients per ward per day can be observed, and observations take place from 9 am until 4 pm.

Physical behavior is defined as the percentage of the total observed time that a patient spent in a specific body position. A distinction will be made between lying, sitting (bedside or chair), and moving (standing, transferring, walking, and cycling). The primary outcome in this study is the percentage of time spent lying.

#### Secondary Outcomes

Secondary outcomes are the percentages of time spent sitting and moving, LOS, and the incidence of immobility-related complications (ie, pneumonia, aspiration, chest infection, pulmonary embolism, deep-vein thrombosis, urinary tract infection, and pressure sores) [31]. LOS and immobility-related complications will be retrospectively retrieved out of the electronic patient file.

### Patient Characteristics

Demographic characteristics that will be documented are gender, age, admission reason, specialism, the use of mobilization tools (ie, rollator, walker, crutches, or stick), urine catheter (yes/no), infusion (yes/no), and main perceived limitations during physical activity (eg, pain and exhaustion). In addition, the health perception and physical functioning of patients will be assessed.

The subjective believed health questionnaire is used to obtain the health perception, defined as “individual’s experience of physical and mental functioning while living his life the way he wants to, within the actual constraints and limitations of individual existence” [32]. The questionnaire consists of 8 questions; question 1 and 2 focus on subjective health, scored on a ladder-type scale from 0 to 10. Question 3 to 8 focus on perceived control and acceptance, scored between 1 (completely disagree) and 7 (totally agree) [33].

The Activity Measure for Post-Acute Care (AM-PAC) is a validated measurement instrument based on the activity limitation domain of the International Classification of Functioning, Disability and Health. In this study, the AM-PAC “6-Clicks” measures of basic mobility and daily activity in acute care will be used. These short forms have shown to be valid for assessing patients’ activity limitations in acute care settings [34,35]. Handgrip strength can indicate the overall strength of an individual and can provide insight into the level of physical function [36,37]. Handgrip strength will be measured with the Jamar hydraulic hand dynamometer, which is an isometric, hydraulic, and easily accessible tool with excellent test-retest reliability (r>0.80) and interrater reliability (r=0.98) [36,37]. The 30-seconds chair stand test is a reliable and valid measurement method for lower extremity strength assessment and a good indicator for a person’s level of physical function [38].

### Sample Size Calculation

In this study, per ward 40 patients will be included per time point. This number is based on earlier studies evaluating physical behavior with the behavioral mapping method [39]. Patients will be included on 4 wards, leading to a total study population of 160 patients. To check if this number is adequate for powered effectiveness analyses, a sample size calculation was performed. For the sample size calculation, unpublished observation data from the UMC Utrecht in 2016 were used, in which 80 patients across the hospital were observed according to the behavioral mapping method. These data demonstrated that patients spent 56.01% of the time lying, with an SD of 32.53. On the basis of an earlier study evaluating the implementation of a multidimensional intervention to improve patients’ physical behavior, a decrease of 15% in the time spent in bed is expected to be feasible [18]. According to the sample size calculation, including a power of 80% and a P value of .05, a sample size of 74 patients would be needed. This confirms that the proposed sample size of 160 patients is more than adequate to evaluate the effectiveness of Hospital in Motion.

### Process Evaluation

**Figure 3:**
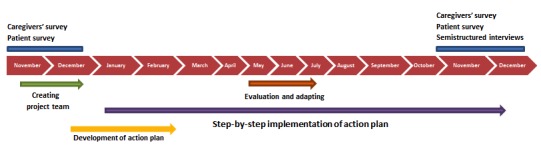
Timeline of process evaluation.

Process evaluations are advised to monitor implementation processes of complex interventions and to evaluate factors of influence on the implementation. In this study, the framework of the medical research council guideline 2008 is followed to guide the process evaluation [40]. The 3 key functions of this framework include implementation, mechanisms of impact, and context. Implementation contains the goals and interventions that have been delivered by the project, including the adaptations, dose and reach, and how this delivery is achieved. The mechanisms of impact include the response (of caregivers and patients) to the interventions, the mediators, and all unexpected pathways and consequences. Context includes all other factors that may affect the implementation, interventions, and outcomes, such as barriers (eg, openness to changes, motivation, workload, and money) and facilitators [40]. For the process evaluation of the Hospital in Motion study on the different wards, a caregivers’ survey, a patient survey, and semistructured interviews with patients and caregivers are developed, which contain items of the 3 key functions of a process evaluation. The caregivers’ survey and the patient survey will be conducted before and after the implementation period. The semistructured interviews will be conducted at the end of the implementation period (Figure 3).

For the caregivers’ survey, questions are formulated focusing on the willingness to change and motivation of the caregivers to help improve patients’ physical behavior. In addition, questions are included to investigate the current state of the 6 topics of the action plan. The scoring of the questions is based on the visual analog scale; a score between 0% and 100% agreement can be given per question. The survey will be sent to all caregivers of the included wards before and after the implementation period.

For the patient survey, the level of encouragement patients perceived from care providers and the environment to be physically active in the past 2 days will be investigated using 6 statements with a 5-point scale. This patient survey will be performed before and after the implementation period. After the implementation, the survey will be supplemented with questions to investigate the success of the implementation of the action plans per ward.

Semistructured interviews with patients and caregivers: After the implementation, semistructured interviews with both patients and caregivers will be undertaken. The interviews will be guided with a topic list based on the 3 key functions of process evaluation as described before [40].

### Statistical Analysis

All statistical analyses will be conducted using IBM SPSS statistics software 25. All outcome variables will be tested on normality with the Kolmogorov-Smirnov test. Patients’ characteristics will be described using descriptive statistics and tested with the Chi-square test, Mann Whitney test, or independent samples t test. Physical behavior is defined as the percentages of the total observed time that a patient spent lying, sitting, and moving. For both the primary outcome (the percentage of time spent lying) and the secondary outcomes (percentage of time sitting and moving), the changes in percentages after implementation will be analyzed. In addition, between-group analyses will be performed per ward. The differences between pre- and postmeasurements will be analyzed with an analysis of covariance, whereby the covariate(s) include baseline variables that may differ between pre- and postmeasurements. If data are not normally distributed, log transformation will be executed before testing.

The process evaluation will be based on the caregivers’ survey, patient survey, and semistructured interviews. Categorical data will be analyzed using Chi-square test and continuous data by using the Mann Whitney test or independent sample t test. To correct for multiple testing, a post hoc multiple comparison test will be performed. The semistructured interviews will be audio recorded and transcribed. Data analysis will follow 3 steps: coding, categorizing, and selecting themes, which will be performed in NVivo 11.

## Results

This study is ongoing. The first participant was enrolled in October 2017 for the premeasurement. The postmeasurements are planned for the end of 2018. The first results are expected to be submitted for publication in autumn 2019.

## Discussion

Despite the evidence about the negative consequences of low levels of physical activity, patients still spend most of the day in bed, leading to unnecessary functional decline and new disabilities in ADL [2,3]. Previous studies demonstrated that increased amounts of physical activity during hospitalization may prevent this functional decline [41]. Furthermore, 3 recent studies reported the results of the implementation of a single intervention to improve physical mobility during hospital stays [14,20,42]. The first study implemented a mobility scale and demonstrated an improved level of physical functioning on a general medicine unit [14]. The second study implemented an enforced mobilization protocol in patients following gastrointestinal cancer surgery and found a reduced number of postoperative pulmonary complications [20]. The third study is a large-scale study in which the implementation of specific mobilization goals (mobilization within 24 hours, mobilization 3 times a day, and progressive and scaled mobility) showed a 10% increase in the frequency of mobilization out of bed [42]. However, to integrate physical activity in usual care, multidimensional approaches with multiple interventions focusing on the whole system are suggested to be more successful [16]. The Eat Walk Engage program of Mudge et al is a good example of a multidimensional approach using multiple interventions, which demonstrated a reduced LOS after the implementation [19]. However, it still remains unclear if physical activity is a modifiable factor during hospital stay.

The Hospital in Motion study has the strength that it contains multiple interventions tailored per ward, developed by a multidisciplinary project team. In addition, it is one of the first known large projects using a multidimensional approach, focusing on the physical environment, caregivers, and patients, instead of only 1 element, to improve physical behavior during hospitalization. Another strength of the Hospital in Motion study is the primary outcome of physical activity. As previous studies mostly included medical outcomes (eg, LOS, remissions, and mortality), levels of physical functioning or frequency of mobilization and the actual amount and change of physical activity have not been evaluated [14,19,20,42]. To get more information about patients’ physical behavior, it is important to assess and evaluate the physical activity levels of patients during hospitalization. For this purpose, the behavioral mapping method is used. This method provides insight into the actual activity level of patients during an average hospital day and also assesses environmental factors such as the people in direct contact with the patient and the patients’ daily activity. This enables detailed evaluation of inpatient physical behavior and differences per ward.

Diverse factors could influence the success of the implementation of Hospital in Motion. The action plan is a multidimensional package of interventions aimed to improve physical behavior. It contains multiple interventions aimed to incorporate physical activity in usual care procedures, targeting the whole care system. This strength is a challenge at the same time. Many factors may affect the implementation process, such as the functioning of the project team, caregivers’ motivation and willingness to change, available time, and perceived workload. The appropriate study design has been discussed extensively within the research team because of the possible influence of confounding factors. As this study primarily aims to integrate physical activity in daily hospital care, more classic research designs (ie, randomized controlled trials) are less suitable. By following a step-by-step implementation process and by performing a process evaluation, the authors will provide useful insights into the changes in usual care and the successful and unsuccessful elements of the implementation process.

## Abbreviations

ADL: activities of daily living
AM-PAC: Activity Measure for Post-Acute Care
HAD: hospitalization-associated disability
LOS: length of stay
UMC Utrecht: University Medical Center Utrecht
